# Successful treatment of severe diffuse alopecia areata with abrocitinib^☆^

**DOI:** 10.1016/j.abd.2024.11.004

**Published:** 2025-04-22

**Authors:** Jinran Lin, Zheng Li, Linxia Shen, Jui-Ming Lin, Ke Tao, Ying Miao, Chunya Ni, Youyu Sheng, Wenyu Wu

**Affiliations:** a Department of Dermatology, Huashan Hospital, Fudan University, Shanghai Institute of Dermatology, Shanghai, China; b Department of Dermatology, Jing’an District Central Hospital, Shanghai, China; c National Clinical Research Center for Aging and Medicine, Huashan Hospital, Fudan University, Shanghai, China

Dear Editor,

Alopecia areata (AA) is a chronic autoimmune disorder characterized by non-scarring hair loss.^1^ Clinically, various patterns of hair loss may be observed. Among them, diffuse AA is a unique subtype, described as widespread scalp hair thinning, which brings a significant psychological burden for the patients.^2^ Systemic treatment for severe AA is limited, but Janus kinase (JAK) inhibitors (baricitinib and ritlecitinib) have recently shown promising results in clinical trials and have been approved by the Food and Drug Administration (FDA) for the treatment of severe alopecia areata.^1^ Other JAK inhibitors, in particular abrocitinib, a highly selective JAK1 inhibitor have been approved for the treatment of moderate-to-severe atopic dermatitis (AD).^3^ but no, clinical trials have been conducted to investigate its theraptherapeutic potential in AA.

In this letter, we report a case series of patients with severe AA who showed improvement following treatment with abrocitinib. Six patients with severe AA, who were treated at our hospital between July 2023 and June 2024, were included in this report. These patients, who had a Severity of Alopecia Tool (SALT) score ≥50, indicating severe AA, either had no response to systemic glucocorticoids or refused their use. They received 100 mg of abrocitinib daily for at least 12-weeks. If the patient has recovered at 12-weeks, the dosing interval is incrementally extended. Patient characteristics were assessed at baseline and SALT scores were measured every 4-weeks.

The patients' average age was 38.4 ± 8.7 years (range, 27–52), with a duration of AA ranging from 1.5 to 6 months (average 3.1 ± 1.7 months). Detailed demographics and clinical characteristics are summarized in Table 1. At baseline, the mean SALT score was 56 ± 7.8 (range, 50–70), and after 8-weeks of treatment, all patients achieved a SALT score ≤20. Two-thirds of the patients (4/6) experienced complete recovery within 12 weeks (SALT score = 0), with an average final SALT score of 2 ± 3.6 (range, 0–9). Table 1 also illustrates the clinical improvements observed during the treatment period. Fig. 1(A‒C) presents representative figures of these patients at baseline and 12-weeks after treatment. Notably, abrocitinib was well-tolerated with no adverse events reported.

**Table 1:** Clinical characteristics and SALT scores of patients.

Patients (no.)	Sex	Age, years	Disease duration, months^a^	IgE level (KUA/L)^b^	Atopic diseases	Initial SALT score	SALT score at week 4	SALT score at week 8	SALT score at week 12	SALT score at week 24
1	F	35	1.5	121	AR	54	36	4	0	5
2	M	27	6	576	AR	60	34	16	9	4
3	F	42	4	47.2	AR	50	14	2	0	3
4	M	41	2	225	AR, AD	70	31	2	1	0
5	F	33	3	136	AR	52	16	4	0	0
6	F	52	2	20.1	AR	50	20	4	0	0

F, Female; M, Male; AR, Allergic Rhinitis; AD, Atopic Dermatitis; SALT, Severity of Alopecia Tool.

a The disease duration is determined from the most recent exacerbation.

b A serum total IgE level >60 KUA/L was defined as elevated.

**Fig. 1:**
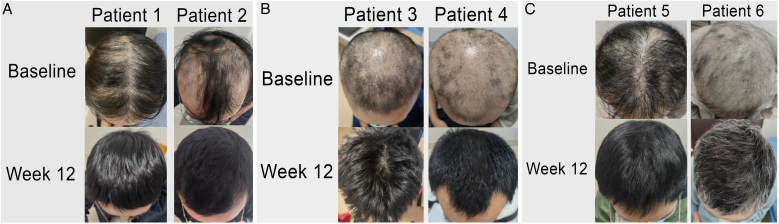
(A‒C) Representative figures of AA patients at baseline and 12-weeks after treatment.

To our knowledge, these are the first clinical experiences evaluating the role of abrocitinib for the treatment of severe diffuse AA, and our patients showed a rapid response to abrocitinib. Past studies have reported successful treatment of abrocitinib in several AA cases with concomitant AD.^4^ Additionally, there is a hypothesis that diffuse AA may be related to hypersensitivity and an increase in serum IgE level.^2^ All six of our six patients had a history of allergic rhinitis and one had moderate AD, where, four of six patients exhibited high serum IgE levels, suggesting that concurrent atopic diseases might predict a positive response to abrocitinib treatment. It is noteworthy to consider that two patients’ conditions worsened after reducing their medication, so further exploration is needed on how to proceed with drug reduction. We advocate for further investigation through randomized controlled trials to assess the efficacy and safety of abrocitinib for severe AA.

## Financial support

Grants from Shanghai Engineering Research Center of Hair Medicine (19DZ2250500), Key Specialty Research Centre of Shanghai Health Commission (2023ZZ02018), Leading Talent Project of Shanghai Health Commission (2022LJ017), Jing'an District Clinical Advantage Special Disease Construction Project (2021ZB01), Clinical Research Plan of SHDC (SHDC22022302), Shanghai Hospital Development Center Foundation (SHDC12024144), Shanghai Municipal Key Clinical Specialty (shslczdzk01002), National Key Research and Development Program of China (2023YFC2509000).

## Authors’ contributions

Zheng Li: Investigation; resources; visualization; writing-original draft.

Linxia Shen: Investigation; resources; visualization; formal analysis.

Jui-Ming Lin: Writing-review & editing.

Ke Tao: Investigation.

Ying Miao: Investigation.

Chunya Ni: Investigation.

Youyu Sheng: Investigation.

Jinran Lin: Funding acquisition; project administration; resources; writing-review & editing.

Wenyu Wu: Conceptualization; project administration; funding acquisition; resources.

## Conflicts of interest

None declared.
